# Does limited virucidal activity of biocides include duck hepatitis B virucidal action?

**DOI:** 10.1186/1471-2334-12-276

**Published:** 2012-10-30

**Authors:** Andreas Sauerbrei, Michael Schacke, Brigitte Glück, Uwe Bust, Holger F Rabenau, Peter Wutzler

**Affiliations:** 1 Institute of Virology and Antiviral Chemotherapy, Jena University Clinic, Friedrich Schiller University of Jena, Hans-Knoell-Strasse 2, Jena, 07745, Germany; 2 Institute of Medical Virology, Hospital of Johann Wolfgang Goethe University of Frankfurt, Paul-Ehrlich-Strasse 40, Frankfurt am Main, 60596, Germany; 3 German Association for the Control of Virus Diseases e.V, Hans-Knoell-Strasse 2, Jena, 07745, Germany

**Keywords:** Duck hepatitis B virus, Vaccinia virus, Disinfectants, Limited virucidal activity

## Abstract

**Background:**

There is agreement that the infectivity assay with the duck hepatitis B virus (DHBV) is a suitable surrogate test to validate disinfectants for hepatitis B virucidal activity. However, since this test is not widely used, information is necessary whether disinfectants with limited virucidal activity also inactivate DHBV. In general, disinfectants with limited virucidal activity are used for skin and sensitive surfaces while agents with full activity are more aggressive. The present study compares the activity of five different biocides against DHBV and the classical test virus for limited virucidal activity, the vaccinia virus strain Lister Elstree (VACV) or the modified vaccinia Ankara strain (MVA).

**Methods:**

Virucidal assay was performed as suspension test according to the German DVV/RKI guideline. Duck hepatitis B virus obtained from congenitally infected Peking ducks was propagated in primary duck embryonic hepatocytes and was detected by indirect immunofluorescent antigen staining.

**Results:**

The DHBV was inactivated by the use of 40% ethanol within 1-min and 30% isopropanol within 2-min exposure. In comparison, 40% ethanol within 2-min and 40% isopropanol within 1-min exposure were effective against VACV/MVA. These alcohols only have limited virucidal activity, while the following agents have full activity. 0.01% peracetic acid inactivated DHBV within 2 min and a concentration of 0.005% had virucidal efficacy against VACV/MVA within 1 min. After 2-min exposure, 0.05% glutardialdehyde showed a comparable activity against DHBV and VACV/MVA. This is also the case for 0.7% formaldehyde after a contact time of 30 min.

**Conclusions:**

Duck hepatitis B virus is at least as sensitive to limited virucidal activity as VACV/MVA. Peracetic acid is less effective against DHBV, while the alcohols are less effective against VACV/MVA. It can be expected that in absence of more direct tests the results may be extrapolated to HBV.

## Background

Hepatitis B is one of the major public health problems worldwide since approximately 350 million, i.e. 5% of the total population, are infected chronically [1]. An important way to prevent infections encompasses disinfection measures to interrupt transmission by the inactivation of the virus on instruments, surfaces and in biological materials [2]. The hepatitis B virus (HBV) can be inactivated effectively inter alia by chemical biocides characterized by broad-spectrum virucidal activity according to the norm EN 14476:2007 [3]. However, the number of agents achieving broad-spectrum virucidal efficacy is limited and they are not required for the inactivation of HBV such as other human blood-borne viruses since these lipophilic viruses with an envelope are characterized by lower stability than viruses without envelope [4]. Thus, a German guideline for testing the virucidal activity of chemical disinfectants in the human medical area [5] differentiates between disinfectants with virucidal activity effective against non-enveloped plus enveloped viruses and disinfectants with limited virucidal activity exclusively effective against enveloped viruses. Limited virucidal activity has been declared when there is a proven efficacy against two representatives of enveloped viruses, the vaccinia virus strain Lister Elstree (VACV) or the modified vaccinia Ankara strain (MVA) [6] and the bovine viral diarrhea virus (BVDV) strain NADL. Even though biocides with limited virucidal activity can be assumed to be effective against HBV, an efficacy cannot be predicted reliably and, therefore, the virucidal efficacy against HBV has to be validated by the use of robust laboratory methods.

So far, the virucidal efficacy of biocides against HBV can be stringently determined only *in vivo* by chimpanzee infection assays [7] with less sensitivity or by the use of primary hepatocyte cultures derived from Tupaias, small-squirrel-like animals living in Southeast Asia [8]. However, while animal protection and economic reasons prohibit the use of higher primates for routine tests on commercial products, the Tupaia model is expensive, the availability of hepatocytes is limited and the test requires human sera with high viral load. Furthermore, surrogate assays for measuring integrity of viral DNA [9], activity of viral DNA polymerase [10], reactivity of HBV surface antigen [11] and the physical integrity of infectious viral particles [12] do not correlate reliably with the infectivity of HBV. In addition, the use of the hepatoma cell line HepG2 [13] is very doubtful. However, redifferentiated Hepa RG cells [14] are well accepted and reproducible as HBV infectivity system, but they have not been applied for testing the hepatitis B virucidal activity of biocides. Thus, the most promising and feasible assay is at present the use of a taxonomically related surrogate virus of the same virus family *Hepadnaviridae*, namely, the duck hepatitis B virus (DHBV), which can be propagated in ducklings or in primary duck embryonic hepatocytes [15,16]. Duck hepatitis B virus shares many physical properties with the closely related HBV [17] and similar inactivation kinetics by disinfectants compared with chimpanzee transmission studies of HBV have been reported [7]. In Germany, the infection of primary duck embryonic hepatocytes with DHBV was established and evaluated for virucidal testing in several studies [18-20].

The objective of the present study was to compare the limited virucidal activity of different biocides, which are often used as ingredients in disinfectants, against DHBV and VACV/MVA. The results should provide information whether the virucidal activity of biocides against VACV/MVA, the most important model viruses for the determination of limited virucidal activity, includes the inactivation of DHBV.

## Methods

### Cell culture

Primary duck embryonic hepatocytes were prepared from liver tissue as described previously [18-20]. Briefly, fertilized Peking duck eggs obtained from the poultry farm Pötzsch (Ostrau, Germany) were incubated at 37.8°C for 21 days. The liver tissue was harvested from 6–7 embryos under sterile conditions, pooled, minced and washed subsequently with phosphate-buffered saline (PBS) supplemented with 1 mg/ml glucose. To ensure the absence of DHBV in the source tissue, the first wash fluid containing blood and liver cells was examined for DHBV DNA by using a qualitative polymerase chain reaction technique [18]. Thereafter, the liver tissue was digested with stirring in the dark using a solution comprising of one volume 0.05% trypsin plus 0.02% ethyl diamine tetraacetate (EDTA) solution (Invitrogen, Karlsruhe, Germany) and 4 volumes PBS plus 1 mg/ml glucose and the resulting cell suspension was removed every 30 min. By the addition of 5% fetal calf serum (FCS, PAA, Pasching, Austria), digestion was inhibited and the cells were collected by centrifugation. After re-suspension in growth medium, cells were stored at 4°C. Digestion of the remaining tissue was continued using fresh trypsin/EDTA solution.

DHBV-negative cells were seeded into 24-well culture plates (CellBIND®, Corning, Acton, United States) with a seeding density of about 0.8 × 10^6^ cells per ml and 1 ml per well. The growth medium consisted of Williams medium E (Invitrogen) supplemented with 1.5% Me_2_SO (Sigma-Aldrich, Taufkirchen, Germany), 15 mM HEPES (Invitrogen), 1% insulin-transferrin-selenium solution (Invitrogen), 10 μM hydrocortisone 21-hemisuccinate (Sigma–Aldrich), 1 mg/ml bovine serum albumin (Sigma-Aldrich), 2 mM L-glutamine (Lonza, Verviers, Belgium), 0.1 μg/ml fungizone (Lonza), 100 U/ml penicillin (Lonza) and 100 U/ml streptomycin (Lonza). For cultivating, cells were incubated at 37°C in a humidified atmosphere containing 5% CO_2_ and the growth medium was replaced after 24 h and on day 4.

Experimental investigations on embryonated hens’ or ducks’ eggs are, in general, regarded as borderline trials between *in vivo* and *in vitro* systems and do not conflict with ethical and legal aspects of animal protection [21]. Therefore, the experiments of this study are not subject to the “Animal Research: Reporting *In Vivo* Experiments” (ARRIVE) guidelines and ethical approval was not necessary.

### Viruses

DHBV-containing serum from congenitally infected ducks was kindly provided by Prof. M. Nassal (University Hospital Freiburg, Department of Internal Medicine II/Molecular Biology, Freiburg, Germany). Sera contained between 10^6.0^ and 10^7.13^ tissue culture infective doses 50% (TCID_50_) of DHBV per ml corresponding to 10^8.77^ and 10^9.90^ DHBV genomic copies. After preparation of 0.5-ml aliquots, sera were stored at −80°C.

### Biocides

Following biocides were used in this study: (i) ethanol (30%, 40%, 50%, 60%, v/v; contact time: 1 min, 2 min), (ii) isopropanol (20%, 30%, 40%, v/v; contact time: 1 min, 2 min), (iii) peracetic acid (0.001%, 0.005%, 0.01%, 0.05%, v/v; contact time: 1 min, 2 min), (iv) glutardialdehyde (0.05%, 0.1%, w/w; pH 8.4; contact time: 0.5 min, 2 min, 5 min), (v) formaldehyde (0.7%, w/v; pH 7.0; contact time: 5 min, 30 min, 60 min). The pH values of the biocides were measured if necessary.

### Preparation of test samples

Test samples for the proficiency panel (stock solutions) were generated by an independent provider (kindly provided by Dr. F. von Rheinbaben, Ecolab Deutschland GmbH, Düsseldorf, Germany). All samples were sent out by a commercial parcel service and arrived 24 h of dispatch.

### Determination of hepatocytotoxicity

In order to determine hepatocytotoxicity, biocides were serially diluted tenfold in growth medium up to a dilution of 10^-8^. One part by volume of water of standardized hardness instead of DHBV-positive serum was mixed with one part by volume of FCS as organic load and eight parts by volume of the biocide. Aliquots of 100 μl from each test concentration and each dilution were then inoculated into two wells of a 24-well micro-titer plate containing monolayer of primary embryonic duck hepatocytes plated 4 to 5 days before. The cells were incubated at 37°C for 7–10 days and examined as described in the chapter “*Immunofluorescent DHBV antigen staining*”. Cytotoxicity was evident when cell monolayers were detached from the growth surface, hepatocytes became round and lost their typical polygonal morphology.

### Quantitative suspension test

Tests were carried out in accordance with the DVV/RKI guideline including minor modifications [5]. One part by volume of DHBV-containing duck serum and one part by volume of FCS were mixed with eight parts by volume of the biocide in a 1.25 fold pre-dilution of the concentration to be tested. The tests were carried out in ambient temperatures of 20-22°C. At the end of the chosen exposure time, the test compounds were removed immediately from the mixture of virus and test formulation by the use of MicroSpin^TM^ S-400 HR Columns (GE Healthcare UK Limited Little Chalfont, Buckinghamshire, UK). To this end, 125 μl of the test mixture was added to the MicroSpin^TM^ column and centrifuged at 737 g for 1 min. Subsequently, 120 μl of the eluted suspension was mixed with ice-cold cell culture medium to obtain a virus dilution of 10^-1.7^ and to prepare serial ten-fold dilutions up to 10^-7.7^. Without delay, 500 μl of each were seeded in one well of 24-well micro-titer plates containing monolayers of primary duck embryonic hepatocytes at day 4 of cultivation and 50 μl cell culture medium. This resulted in final viral dilutions from 10^-2^ to 10^-8^. Afterwards, the cells were incubated at 37°C for 7–10 days.

### Immunofluorescent DHBV antigen staining

Indirect immunofluorescent antigen staining was employed for detection of DHBV surface antigen as described previously [18,19]. In short, the cells were washed once in PBS and fixed with acetone/methanol (1:1) at −20°C for 10 min. After air drying, cells were re-hydrated in PBS for 5 min and incubated with polyvalent rabbit anti-DHBs (kindly provided by Dr. D. Glebe, Institute of Medical Virology, National Reference Centre for Hepatitis B and D, Justus Liebig University, Giessen, Germany) antiserum diluted 1:100 for 1 h at 37°C, washed three times whith PBS and stained with goat anti-rabbit IgG Alexa Fluor® 488-labeled antibody (Life Technologies, Darmstadt, Germany) diluted 1:400 in PBS containing 0.001% Evans blue and Tween 80. Stained cells were analyzed using the fluorescent microscope Nikon DIAPHOT-TMD (Nikon-Corporation, Tokyo, Japan). The total number of positive cells per well was estimated visually.

### Statistical analysis

Each experiment was performed at least eight times, maximally twelve times. From the total number of positive cells per well (maximally 40% infected cells), the virus titers of each test and the corresponding virus control (not treated with biocide) were calculated. Titer reduction is presented as the difference between the control virus titer and the virus titer after contact time with the biocide. This difference is given as reduction factor (RF) including its 95% confidence interval (CI). A reduction of infectivity of ≥4 log_10_ steps (inactivation ≥99.99%) was regarded as evidence of sufficient virucidal activity. The calculation was performed most widely according to the DVV/RKI guidelines [5].

## Results

The detailed results of virucidal testing of the five different biocide ingredients against DHBV are summarized in Tables 1 and 2. For comparison, these tables contain the results of testing these biocides against VACV and MVA published previously by Rabenau et al. [6]. After exposure of DHBV to 40%, 50% and 60% ethanol, ≥4 log_10_ reductions in viral titers were achieved after time intervals of 1 min and 2 min. By contrast, the use of 30% ethanol was associated with RFs of 2.99 ± 0.78 after 1-min and 3.46 ± 0.64 after 2-min exposure. These findings corresponded widely to the inactivation of VACV and MVA, but a sufficient activity was detected for 40% ethanol within 2-min exposure. The use of 20% isopropanol resulted in RFs for DHBV within 1.96 ± 0.66 after 1 min and 2.33 ± 0.61 after 2 min. Whereas 30% isopropanol after 1-min exposure did also not result in sufficient activity (RF 3.39 ± 0.88), there was sufficient activity when 30% isopropanol was used for 2 min and 40% isopropanol for at least 1 min. In comparison, 40% isopropanol for at least 1 min was necessary to inactivate VACV/MVA. Peracetic acid at a concentration of 0.01% inactivated the infectivity of DHBV after 2-min exposure as was the case for 0.05% peracetic acid after 1 min and 2 min. No sufficient efficacy against DHBV was seen when peracetic acid was used at concentrations of 0.001% and 0.005% after 1 min and 2 min as well as for 0.01% after 1-min exposure (RF between 1.36 ± 0.35 and 3.55 ± 0.60). These findings were different from those with VACV/MVA whose infectivity was already inactivated significantly by the use of 0.005% peracetic acid after 1 min. The hepatocytotoxicity of ethanol and isopropanol at the concentrations tested was <2.0 log_10_ TCID_50_ of DHBV whereas the hepatocytotoxicity of peracetic acid ranged between <2.0 (at concentrations of 0.001 – 0.005%) and <3.0 log_10_ TCID_50_ (at concentrations of 0.01 – 0.05%).

**Table 1:** **Limited virucidal activity of ethanol**, **isopropanol and peracetic acid against duck hepatitis B virus** (**DHBV**) **depending on concentration and contact time in comparison to vaccinia virus** (**VACV**)/**modified vaccinia Ankara strain** (**MVA**)

**Biocide**	**Concentration (%)**	**Contact time** (**min**)	**Reduction factors**	
			**DHBV**	**VACV**/**MVA**
Ethanol	30	1	2.99 ± 0.78	−0.10 ± 0.29/0.01 ± 0.26
		2	3.46 ± 0.64	0.32 ± 0.28/-0.01 ± 0.30
	40	1	≥**4**.**35**	≥**3**.**28**/2.94 ± 0.27
		2	≥**4**.**38**	≥**4**.**59**/≥**4**.**05**
	50	1	≥**4**.**84**	≥**5**.**11**/≥**4**.**76**
		2	≥**4**.**88**	≥**5**.**11**/≥**5**.**76**
	60	1	≥**4**.**88**	≥**5**.**11**/≥**5**.**76**
		2	≥**4**.**88**	≥**5**.**11**/≥**5**.**76**
Isopropanol	20	1	1.96 ± 0.66	0.01 ± 0.29/0.08 ± 0.25
		2	2.33 ± 0.61	−0.01 ± 0.30/-0.04 ± 0.30
	30	1	3.39 ± 0.88	1.63 ± 0.30/1.35 ± 0.22
		2	**4**.**10** ± **0**.**80**	1.87 ± 0.26/2.67 ± 0.27
	40	1	≥**4**.**00**	≥**5**.**11**^1^/≥**4**.**76**^1^
		2	≥**4**.**00**	≥**5**.**11**^1^/≥**4**.**76**^1^
Peracetic acid	0.001	1	1.36 ± 0.35	0.74 ± 0.31/0.74 ± 0.27
		2	1.67 ± 0.41	1.62 ± 0.30/1.79 ± 0.30
	0.005	1	2.43 ± 0.51	≥**4**.**50**/≥**5**.**66**
		2	3.05 ± 0.39	≥**4**.**50**/≥**5**.**66**
	0.01	1	3.55 ± 0.60	≥**4**.**48**/≥**4**.**85**
		2	≥**4**.**08**	≥**4**.**48**/≥**4**.**85**
	0.05	1	≥**4**.**08**	≥**4**.**15**/≥**4**.**35**
		2	≥**4**.**08**	≥**4**.**15**/≥**4**.**35**

Results for VACV and MVA are given as means calculated from the results of three different laboratories (Rabenau et al. [10]).

“≥” - Determination of RF is limited by hepatocytotoxic reactions and/or the amount. Calculation of 95% confidence interval is not possible.

^1^ Results are only from one laboratory.

**Table 2:** **Limited virucidal activity of glutardialdehyde and formaldehyde against duck hepatitis B virus** (**DHBV**) **depending on concentration and contact time in comparison to vaccinia virus** (**VACV**)/**modified vaccinia Ankara strain** (**MVA**)

**Biocide**	**Concentration** (%)	**Contact time** (**min**)	**Reduction factors**	
			**DHBV**	**VACV**/**MVA**
Glutardialdehyde	0.05	0.5	2.79 ± 0.78	1.62 ± 0.25/1.14 ± 0.29
		2	≥**4**.**05**	≥**4**.**35**/≥**3**.**82**
		5	≥**4**.**05**	≥**4**.**35**/≥**3**.**82**
	0.1	0.5	≥**4**.**05**	2.91±0.22 / 2.21±0.25
		2	≥**4**.**05**	≥**4**.**35** / ≥**3**.**82**
		5	≥**4**.**05**	≥**4**.**35** / ≥**3**.**82**
Formaldehyde	0.7	5	2.00 ± 0.40	1.15 ± 0.28/≥**2**.**09**
		30	≥**3**.**06**	>**2**.**62**/>**2**.**14**
		60	≥**3**.**08**	>**3**.**16**/>**2**.**14**

Results for VACV and MVA are given as means calculated from the results of three different laboratories (Rabenau et al. [10]).

“≥” - Determination of RF is limited by hepatocytotoxic reactions and/or the amount. Calculation of 95% confidence interval is not possible.

Glutardialdehyde showed a sufficient efficacy against DHBV corresponding to ≥4 log_10_ reduction in viral titers when it was used at concentration of 0.05% for ≥2 min and at concentration of 0.1% for ≥0.5 min. By contrast, 0.5-min exposure of 0.05% glutardialdehyde resulted in RF of 2.79 ± 0.78. These results were in absolute concordance to VACV/MVA even though ≥4 log_10_ titer reduction could not always be achieved because of the high cytotoxicity of this biocide. This also applies to 0.7% formaldehyde which did not inactivate the infectivity of DHBV and VACV/MVA within 0.5-min exposure, but resulted most likely in sufficient activity after at least 30-min exposure. At the concentrations tested, the hepatocytotoxicity of glutardialdehyde was <3.0 and of formaldehyde <4.0 log_10_ TCID_50_ of DHBV.

## Discussion

In principle, enveloped viruses are considered to be relatively sensitive to biocides. However, validation of the virucidal action of disinfectants against HBV is essential since the hepatitis B virucidal activity of biocides cannot be predicted and the human blood plasma protects the virus from inactivation [22]. In particular, Payan et al. [13,23] have described HBV as an enveloped virus that may be less easy to inactivate. In Germany, a national test method has been published for the determination of limited virucidal activity [5] defined as active against enveloped viruses such as HBV, human immunodeficiency virus and hepatitis C virus (HCV) [24]. Unfortunately, feasible HBV infectivity assays have not yet been established for testing virucidal activity of biocides. Therefore, surrogate infectivity assays based on DHBV have been recommended to make a label claim of product efficacy to hepadnaviruses by the Environmental Protection Agency in the United States [25] and in Australia [26,27]. However, the DHBV model has also several disadvantages that prevent its use in virological laboratories. The model established in Germany [18] requires hepatocytes from DHBV-negative duck embryos for *in vitro* infection. By contrast, the virus has to be obtained from serum of ducks infected congenitally or experimentally. This is an unreliable source and no reasonable basis for standardization. Furthermore, the DHBV as all hepadnaviruses is non-cytopathogenic and additional methods such as the indirect immunofluorescent antigen staining are required to verify replication of DHBV in inoculated hepatocytes. That is why information is necessary if testing of biocides against DHBV is essential and should be included in several guidelines or whether disinfectants with the label claim limited virucidal activity according to the German DVV/RKI guideline can be regarded as effective against DHBV.

The present study compared the limited virucidal activity of five different biocides, which are often used as ingredients for commercially available disinfectants, against DHBV and VACV/MVA. The latter represent the classical test virus for the determination of limited virucidal activity. The data for VACV/MVA have been published recently to replace VACV by MVA because of the potential risk of laboratory-acquired infections caused by VACV [6]. To ensure the comparability of all data, the test samples used in both studies were generated by the same provider and the quantitative suspension tests were carried out most widely in accordance with the DVV/RKI guideline [5]. The concentrations of the biocides and the exposure times were chosen to see a kinetic of virus inactivation and a shift from non-effective to effective concentrations and exposure times. All tests were performed with a protein load of 10% FCS. Sufficient activity was shown by a virus titer reduction of at least 4 log_10_ with the exception of formaldehyde that resulted in highest cytotoxicity. In most studies reported in the literature, a compound was considered effective against DHBV when it reduced the viral titer by at 3 log_10_ units [26,28]. As the results of this study show, all basic biocides such as alcohols, peracetic acid, glutardialdehyde and formaldehyde inactivated DHBV and VACV/MVA at relatively low concentrations and within short exposure times. Against ethanol and isopropanol, a marginally higher susceptibility of DHBV could be detected. By contrast, the DHBV was more stable against peracetic acid than VACV/MVA. Gutardialdehyde and formaldehyde showed a comparable virucidal activity against both the DHBV and VACV/MVA. Limited unpublished results with HBV and the use of Tupaia cells correspond widely with these findings (personal communication: D. Glebe, Institute of Medical Virology, National Reference Centre for Hepatitis B and D, Justus Liebig University, Giessen, Germany). Thus, the data presented for DHBV are most likely also valid for HBV.

In addition to VACV/MVA, the BVDV has been recommended as second test virus for the determination of limited virucidal activity of disinfectants according to the German DVV/RKI guideline. Although the great susceptibility of BVDV, the surrogate of HCV, against biocides has not initiated extended studies, preliminary data suggest that this enveloped virus has a comparable if not a lower stability than VACV to biocides [29,30]. Comparable to the data presented in this study, the suitability of VACV and BVDV for determining activities of alcohol-based hand rubs against clinically relevant enveloped human viruses such as herpes simplex virus type 1 and type 2 as well as human and avian influenza A viruses has been reported in the literature [31].

## Conclusions

Biocides which are effective against VACV/MVA, the most important model viruses for the determination of limited virucidal activity, show a reliable inactivation of DHBV infectivity. While peracetic acid is less effective against DHBV, alcohols are less effective against VACV/MVA. However, the findings show that DHBV and VACV/MVA exhibit a comparable tenacity. This means that the validation of disinfectants against DHBV can be omitted in practice when VACV or MVA have been included. It can be expected that in absence of more direct tests the results may be extrapolated to HBV.

## Competing interests

The authors declare that they have neither financial nor non-financial competing interests.

## Authors’ contributions

AS: author of the publication, also provided analysis and interpretation of data, responsible for study design. MS: co-author of the publication, carried out the preparation of primary duck embryonic hepatocytes. BG: co-author of the publication, carried out the harvest of liver tissues from duck embryos. UB, HR and PW: co-authors of the publication and responsible for study design. All authors have read and approved the final manuscript.

## Source of support

The study was supported by the German Association for the Control of Virus Diseases e.V. (DVV).

## Pre-publication history

The pre-publication history for this paper can be accessed here:

http://www.biomedcentral.com/1471-2334/12/276/prepub
